# Narrative sozioökonomischer Ungleichheit. Ein Überblick

**DOI:** 10.1007/s41358-022-00313-7

**Published:** 2022-03-14

**Authors:** Christopher Smith Ochoa, Taylan Yildiz

**Affiliations:** 1 NRW School of Governance, Institut für Politikwissenschaft, Universität Duisburg-Essen, Lotharstraße 53, 47057 Duisburg, Deutschland; 2 Forschungsinstitut Gesellschaftlicher Zusammenhalt (FGZ), Goethe-Universität Frankfurt am Main, Frankfurt am Main, Deutschland

**Keywords:** Erzählung, Verteilungsfrage, Sozialpolitik, Ungleichheitsdiskurs, Sprache, Storytelling, Economic distribution, Social policy, Inequality discourse, Language

## Abstract

Der Band versammelt neuere Beiträge der sozialwissenschaftlichen Ungleichheitsdiskussion. Ausgangspunkt ist die Überzeugung, dass Ungleichheit nicht nur einen *sozialen Zustand* darstellt, den es quantitativ zu erklären gilt, sondern auch einen *Erfahrungsgegenstand *markiert, der verstanden werden muss. Dieses Motiv zentriert sich um den Begriff des Narrativs, der neuerdings auch in der Ökonomie immer mehr dazu genutzt wird, die wirklichkeitskonstruierende Kraft der Sprache in den Blick zu bekommen. Der Band ist in drei Teile gegliedert. Zunächst wird das Problem der Validität des Erzählens aus der Perspektive der Datenforschung behandelt. Im zweiten Teil kommen Beiträge zu Wort, die sich mit erzählerischer Legitimitätsbildung auseinandersetzen, bevor der abschließende Teil dann mit empirischen Untersuchungen in diesem Feld abschließt.

## Abstract (en)

This volume features contributions on the discussion in the social sciences on inequality. It is based on the premise that inequality is not only a quantifiable *material state* but also a *lived* *experience* which must be understood. It accordingly centers on narrative; a concept which has garnered much attention among renowned economists seeking to unpack the reality-creating power of language. The volume comprises three parts. First, validity problems in narratives are discussed from the perspective of empirical data research. Second, contributions grapple conceptually with the legitimacy-creating capacity of narratives, which is, third, explored in empirical studies.

## Einleitung

Die Verteilung knapper und zugleich wertvoller Güter gehört zu den Grundfragen moderner Gesellschaften. Mit ihnen werden nicht nur Konsummöglichkeiten verteilt, sondern zugleich auch Lebenschancen, die für eine angemessene Teilhabe am sozialen und politischen Leben elementar sind (Groh-Samberg 2009; Kreckel 2004; Hradil 2001). Wie allerdings eine „Politik der Lebenschancen“ erreicht werden kann, die „möglichst alle Menschen in die Lage versetzt (…), ihr Leben autonom erfolgreich zu gestalten“ (Mau 2012, S. 219), ist eine offene Frage, kontrovers und emotionsbeladen. Unter anhaltenden oder gar steigenden Verteilungskonflikten erweist sie sich bisweilen als unlösbar. Zwar mangelt es weder der Autonomitätsnorm noch der Einsicht in die Notwendigkeit der materiellen Grundsicherung an allgemeiner Akzeptanzfähigkeit. Ebenso gehört auch die Forderung nach möglichst robusten Aufstiegskanälen zum Standartrepertoire marktliberaler Diskursordnungen. Das führt aber nicht dazu, dass sich Ungleichheitsfragen umstandslos auf das Terrain technisch lösbarer Problembeschreibungen herunterformatieren könnten. Eher scheint das Gegenteil der Fall. Nach wie vor besteht eine ausgeprägte Umstrittenheit sowohl hinsichtlich der Faktenlage von Ungleichheit als auch den politischen Schlussfolgerungen, die auf der Ebene des staatlichen Handelns zu ziehen wären. Zudem ist keineswegs gewiss, wo sich die sozialen Kippmomente befinden, in denen Ungleichheit eine Politik der Lebenschancen unmöglich macht und die negativen Folgewirkungen eines Marktversagens nicht mehr zu kompensieren erlaubt (Sandel 2020; Smith Ochoa 2020, 2022). Bei aller Übereinkunft über die allgemeinen Zielvorgaben bleibt das Aufgabenspektrum der Sozialpolitik also vertrackt und die marktliberalen Demokratien kommen mit ihren eigenen Versprechen immer wieder ins Hadern. Womöglich liegt es daran, dass sich „Ungleichheitspolitik“ als eine Art Aufräumarbeit präsentiert, die auf schwierigem Posten um Beliebtheit ringt.

Dabei unterliegt die Debatte konjunkturellen Schwankungen. Eine intensive Beschäftigung mit ihrer Dynamik könnte einiges über die (noch) unrealisierten Möglichkeiten in dem Politikfeld aussagen. So ist die Frage der sozialen Ungleichheit etwa nicht dauerhaft präsent, sondern wird mal intensiver, mal weniger intensiv gestellt und es sieht so aus, als ob sich ihr politischer Impetus besonders im Kontext der Erhebung und Veröffentlichung empirischer Ungleichheitsdaten entwickeln würde. Zahlen scheinen hier mehr als in anderen Politikfeldern die zentralen Diskurstreiber zu sein (Smith Ochoa und Yildiz 2019). Das dürfte daran liegen, dass Zahlen generell die Aufgabe zugeschrieben wird, zu *objektivieren *und die empirische Ungleichheitsforschung deshalb dazu neigt, ihre epistemische Unsicherheit über den Zustand der Ungleichheit durch statistische Aussagen und mathematische Berechnungen zu reduzieren.^1^ Und da die Forschung ebenso wenig wie die politischen Interpreten der Daten Schwierigkeiten darin haben, einhellig darüber zu befinden, ob sich die epistemische Unsicherheit auf dem Wege statistischer Berechnungen allein tatsächlich beheben lässt (Beckert 2018), brechen bei aller Normkonformität immer wieder Normkonflikte auf; nicht so sehr als Wertekonflikte, sondern eher in der Form datenbezogener Deutungskonflikte. Was die statistischen Befunde sagen, ob sie etwas vergessen oder einen ungleichheitsrelevanten Gegenstand falsch erfasst haben oder welche Handlungsaufforderungen aus dem erreichten Stand des Wissens zu ziehen sind, entziehen sich dem Objektivitätsverständnis der empirischen Forschung und markieren einen Fall von *Politizität*.

Die Statistik bildet also nicht nur ab. Sie animiert auch den Gegenstand, ohne sicherstellen zu können, dass die Kontroverse in den Bahnen des Faktischen bleibt, in denen sie sich zu bewegen vorgibt, oder nicht auch andere Phänomene diskursstrukturierende Kräfte entwickeln. Aktuell verhandeln wir etwa angeregt durch die globale Banken- und Finanzkrise (2007–2009) sowie Thomas Pikettys *Das Kapital im 21. Jahrhundert* (2014) eine Reihe mehr oder weniger stark ausgeprägter Klagen über eine womöglich rapide wachsende Kluft zwischen Arm und Reich ebenso wie wir eine Reihe eloquenter Rechtfertigungen verzeichnen können, die dagegen auf einen Fahrstuhleffekt verweisen. Auf der einen Seite wird die Multiplikationsfähigkeit der Ungleichheit problematisiert. So wie Vermögen noch mehr Vermögen nach sich ziehe, so ziehe auch Ungleichheit weitere Ungleichheiten nach, vor allem jene der mangelnden Repräsentation und Teilhabe, was angesichts der COVID-19-Pandemie gar eine biopolitische Qualität erfährt (Deaton 2021; Oxfam 2021; Romei 2020). Auf der anderen Seite wird die Bedeutung der Ungleichheit als eine zentrale Voraussetzung für die korporative Stärke einer Nation herausgestellt, die sich letztlich auf *Alle* positiv auswirke, selbst dann, wenn sich die Verteilungsspielräume selbst wieder ungleich verteilen würden. Das wäre ja im Sinne der erbrachten Leistung auch nur gerecht.

Vor diesem Hintergrund kann sich die Sozialwissenschaft entweder auf die eine oder andere Seite der Debatte schlagen oder versuchen eine datenvermittelte Position zu finden, die diskret dabei hilft, die Diskurslage zu korrigieren und Klärung zu verschaffen. Sie kann sich aber auch, und das ist die Strategie des vorliegenden Heftes, mit den Diskursdynamiken und den sie strukturierenden narrativen Praktiken befassen, um so auch jene schwer in Zahlen zu fassenden Einflussgrößen thematisieren zu können, die sich auf der subjektiven Seite der Problematik konstituieren und die in der empirischen Forschung zu Ungleichheit bislang doch eher vernachlässigt werden. Abgesehen von unserer eigenen Vorliebe für interpretative Forschungen haben uns in diesem Anliegen zwei wichtige neuere Arbeiten motiviert. Das ist zum einen Robert Shillers *Narrative Wirtschaft* (2020), in der ein Nobelpreisträger das Argument entwickelt, dass marktkonforme Ordnungen weniger durch jene rationalen Prozesse gekennzeichnet sind, die die neoklassische Ökonomie im Umgang mit knappen Gütern für dominant hält. Vielmehr sei der Gegenstand Ökonomie durch die Viralität von Geschichten bestimmt. Er funktioniert für Shiller nicht allein durch den Wettbewerb um die Früchte einer reinen Dingwelt, von der sich behaupten ließe, dass dort „ausschließlich Konstanten, Funktionen und stochastische Interaktionen den Ton angeben“ (Boltanski und Esquerre 2018, S. 252). Vielmehr sie in die sprachlichen Gebilde der jeweiligen Gesellschaften und den dort dominierenden Erzählungen eingebettet. Einen weiteren Anlass haben wir in Jens Beckerts *Imaginierte Zukunft* (2018) gefunden, wo der Wirtschaftssoziologe das fiktionale Denken zu einer elementaren Triebkraft des Wirtschaftshandelns erhebt und so die zentralen Diskursdynamiken des Ökonomischen jenseits der Dichotomie von Fakt und Fiktion verortet. Dabei geht es Beckert nicht nur darum, das Faktische als diskursvermittelte Realität auszuweisen, sondern um die Einsicht, dass die positivistischen Tools der Ökonomie Instrumente sind, die nicht nur Wirklichkeitsbilder generieren, sondern auch Legitimierungen schaffen. Die Einleitung wird sich zunächst – ausgehend von diesen beiden Studien – mit der Relevanz des Erzählens für die Ungleichheitsthematik befassen und ihre Rolle als Mittler von Faktizität und Legitimität thematisieren. Danach werden die hier versammelten Beiträge kurz skizziert und ihre Bedeutungen für eine narrative Ungleichheitsforschung herausgestellt.

## Ungleichheit und Narrative

Thomas Piketty eröffnet sein Buch *Kapital und Ideologie* mit einer folgenreichen Feststellung, wonach jede menschliche Gesellschaft ihre Ungleichheiten rechtfertigen können muss, „mitunter auch jenseits aller Vernunft“, wie es dort ausdrücklich heißt (Piketty 2020, S. 50):

Dieser zugleich intellektuellen, institutionellen und politischen Auseinandersetzung entspringen im Allgemeinen eine oder mehrere herrschende Erzählungen, auf die sich die bestehenden Ungleichheitsregime stützen. (Ebd. 2020, S. 13).

Ungleichheit ist für Piketty also zunächst keine „wirtschaftliche oder technologische“, sondern vor allem „eine ideologische und politische“ Erscheinung (2020, S. 21). So nachvollziehbar sein Argument hier auch erscheinen mag, ist es doch ungewöhnlich, dass sich eine fast ausschließlich auf statistische Daten stützende Wissenschaft so zentral auf die Rolle ideologisch vermittelter Rechtfertigungen bezieht und damit jenes Feld des zwischenmenschlichen Lebens in ihren Gegenstandsbereich einbezieht, das in der Ökonomie bislang eher als ihre Negation behandelt wurde. Denn „jenseits aller Vernunft“ ist außerhalb jeglicher Ratio und damit jenem Modus des menschlichen Denkens und Handelns fremd, der als ultimative Referenz des kapitalgetriebenen Wirtschaftens gilt. Aber Piketty’s Ideologiebegriff folgt keiner tieferen Auseinandersetzung mit der Komplexität des Subjekt-Objekt-Verhältnisses. Wer sich eingehender für derartige Spannungen interessiert, ist besser beraten, sich etwa mit den Arbeiten von Joseph Vogl zu befassen (2002, 2021). Piketty steht vielmehr in der Tradition einer Wirtschaftssoziologie, wo die materiellen Zustände durch eine ideelle Überwölbung als diskursiv vor kritischen Zugriffen abgeschirmt gelten^2^. Allerdings gelingt es Piketty mit seiner raffinierten Re-Positionierung des Ideologiebegriffs im Diskurs der Ökonomie für einen Zusammenhang zu werben, der unter dem vielversprechenden Begriff des Rechtfertigungsnarrativs doch überaus produktive Perspektiven auf soziale Grundfragen erlaubt (Fahrmeir 2013). Offenbar hat sich die Begründungsphilosophie trotz der Dominanz des ökonomischen Paradigmas nie wirklich aus den Sozialwissenschaften verabschiedet und umso erstaunlicher ist es, dass sie nun über den Umweg wirtschaftswissenschaftlicher Kontroversen in die politische Debatte zurückkehrt. Vor diesem Hintergrund lässt sich sagen, dass Ungleichheit mit Ideologie, wie Piketty etwa vermutet, nicht nur deshalb zusammenhängt, weil sich die Herrschenden mit einem subjektiven Bindemittel auszustatten versuchten, das ihnen statussichernde Effekte zu erzeugen und zu stärken verspricht. Ungleichheit und Ideologie sind vielmehr auf einer tieferen Ebene des menschlichen Seins verwoben, und zwar dort, wo es um die Bedingungen der Möglichkeit von Rationalität geht und weniger um das weltliche Verlangen nach einem Handlungsskript, das einer Akteur:in auf ihrem umkämpften Weg der Zielerreichung eine im Idealfall bestmögliche Position verschafft. Denn die Fähigkeit des Menschen „sich anhand rechtfertigender Gründe in der Welt zu orientieren“, hängt zunächst mit dem Tatbestand der Komplexität und Sozialität des menschlichen Lebens zusammen, die letztlich *Alle* gleichermaßen erfasst (Forst 2013, S. 11):

Das Vermögen der Vernunft ist die Fähigkeit, für seine Meinungen und für seine Handlungen Rede und Antwort stehen zu können (…). Dieses Rede-und-Antwort-Stehen ist eine soziale Praxis kulturell und historisch situierter Wesen, die einerseits frei sind, ihre Gründe zu wählen und zu prüfen, andererseits aber daran gebunden, welche Gründe ihnen zur Verfügung stehen und welche als gut oder rechtfertigend gelten. Der Raum der Gründe ist ein Raum der Rechtfertigungen, der nicht nur Einzelhandlungen, sondern auch komplexe Handlungsordnungen, also soziale Verhältnisse und politische Institutionen, legitimiert (Forst 2013, S. 11 in Anlehnung an Wilfried Sellars).

Die Rückkehr zum *Raum der Gründe* bahnt sich auch an anderer Stelle an. Wie Piketty, der diesen Raum nicht an den Grenzen der Vernunft enden sieht, schlägt ja auch Shiller vor, die sozioökonomischen Fragen der Gegenwart nicht mehr nur mittels der mathematischen Analyse von Makrodaten zu beantworten, sondern sie ebenso gut aus der Perspektive der normativen Kontroversen zu betrachten, in die sie eingebettet sind. Er beklagt, dass sich die Ökonomie als Disziplin so sehr von den szientistischen Prämissen des Positivismus abhängig gemacht habe, dass sie erst gar kein Interesse daran entwickeln könnte, die konstituierenden Narrative ihres Gegenstandes zu studieren, geschweige denn sich mit den Ungleichheitsideologien zu befassen, auf die Piketty abhebt (Shiller 2020, S. 38, 39). Zwar ist dem Fach in seiner eigenen wissenschaftstheoretischen Reflexion durchaus bekannt, dass ohne Narrative selbst einfache Dateninterpretationen kaum möglich sind (Schnitzler 2017, S. 73), aber die wirklichkeitskonstruierende Kraft eines „Rede-und-Antwort-Stehens“, das sich in einer Welt realer Wirtschaftssubjekte vollzieht, bleibt meist seltsam unbehandelt. Diesen Verkürzungen der rationalistisch-naturalistischen Grundtendenz der Ökonomie versucht Shiller nun durch einen „revolutionären Ansatz“ entgegenzutreten. Sein Konzept der narrativen Wirtschaft soll das journalistische Schaffen und Rezipieren wirtschaftsrelevanter Texte als einen zentralen Ort des Ökonomischen zu verstehen helfen und die Wirtschaftswissenschaften so mit einem Verständnis für letztlich unberechenbare, kontingente Brüche versorgen. Es seien ja die rekursiven Erzählungen von Ungleichheit und das Rechtfertigen der Konzentration von Macht und Vermögen, die insbesondere in Krisenzeiten einen „kausalen Effekt“ auf die Wirtschaftsprozesse eines Landes ausübten: „Neue ansteckende Narrative verursachen ökonomische Ereignisse und ökonomische Ereignisse verändern Narrative.“ (Shiller 2020, S. 131).

Ein etwas anders gelagertes Plädoyer für die Berücksichtigung narrativer Elemente legt Jens Beckert vor. Dabei beschränkt er sich nicht auf die ontischen Fragen des Wirtschaftens, also auf die narrative Dynamik oder gar Seinsweise ökonomischer Prozesse. Vielmehr interessieren ihn epistemische Fragen, also Fragen, die sich damit befassen, wie sich die Disziplin selbst mit fiktionalen Wissenstechniken ausstattet und im Gewand mathematischer Berechnungen auf jene Wirklichkeit einwirkt, die sie objektiv darzustellen vorgibt. Dabei geht es nicht nur um das Problem der Dateninterpretation, sondern um das Problem der Datenintervention. Wenn Piketty und Shiller im Erzählen eine wirtschaftsrelevante Größe sehen, die sich im journalistisch vermittelten und nicht selten ideologisch imprägnierten Austausch ökonomischer Wirklichkeitsbilder studieren lasse, dann weist uns Beckert darauf hin, dass auch die empirische Arbeit der Ökonomie einen wesentlichen Anteil am Bau jener „Luftschlösser“ hat, die im politischen Wettbewerb der Narrative einander entgegentreten. Er sieht die Wirtschaftswissenschaften also selbst als ein in den „Raum der Gründe“ eingezogenes Unternehmen, die Shiller sich in seiner narrativen Wirtschaft lediglich auf eine andere Art zu studieren vornimmt.

Damit fügen sich beide Perspektiven zu einer Art „doppelten Hermeneutik“ des Ökonomischen, die sich wahrscheinlich mit Pierre Rosanvallons (2017) Demokratietheorie noch am sinnvollsten verknüpfen und politikwissenschaftlich weiterdenken lassen. Eine solche Verknüpfung ließe sich in aller gebotener Kürze etwa folgendermaßen formulieren: Sollte Ökonomie, wie Shiller argumentiert, narrativ verfasst sein, können Wirtschafts- und Sozialdaten kein objektives Bild vom Gegenstand verschaffen. Sie würden, wie Beckert zeigt, zum Bestandteil einer politischen Arithmetik werden, die jene repräsentativen Mechanismen überfordert, die Rosanvallon auf der formalen Seite demokratischer Ordnungen verbucht. Politische Repräsentationslücken und damit einhergehende Unzufriedenheiten wären die Folge, wenn sie es nicht schon sind. Insofern wären Demokratien mit Rosanvallon auch gut beraten, eine narrative Form der Repräsentation auszubilden, also eine isomorphe Beziehung zum Gegenstand Ökonomie einzugehen, wie er u. a. von Shiller und Beckert beschrieben wird. Das könnte verhindern, dass sich die Ungleichverteilung von knappen und zugleich wertvollen Gütern zum unlösbaren politischen Problem entwickelt, zumindest dann, wenn es zugleich auch gelingt, die narrative Repräsentation normativ an das Ideal der gegenseitigen Anerkennung und Wertschätzung zu binden, wie es Rosanvallon vorschlägt.

Diese hier nur kurz skizzierten Zusprüche für eine sozialwissenschaftliche Narratologie deuten bereits auf ein mögliches Assoziationsniveau, auf dem sich unterschiedliche Forschungsmotive in den Dienst eines gemeinsamen Studiums der wirklichkeitskonstruierenden Relevanz des Erzählens stellen ließen. Was wir hier allerdings beabsichtigen, ist keine umfassende Konvergenzleistung, sondern eine um das Thema Ungleichheitserzählungen kreisende Konturierung des Forschungsfeldes. Damit sind nicht nur konzeptionelle Debatten zu berücksichtigen, sondern auch ereignisinduzierte Gründe zu besprechen, die sich mit der Hinwendung zum Erzählen in Verbindung bringen lassen. Auf einer abstrakten Ebene lassen sich solche Gründe in den Rahmen einer Ereignisklasse fassen, die unter dem Begriff des postfaktischen Zeitalters steht. Postfaktizität, selbst eine Verfallsgeschichte, bringt in Überzeichnung negativer Aspekte eine Erfahrung zum Ausdruck, die mit der mangelnden Reichweite der Rationalitätsvorstellungen und Objektivitätsbilder der Moderne zusammenhängt. Und dies lässt sich auf beiden Seiten der doppelten Hermeneutik nachspüren. Auf der Seite der ontischen Feststellung etwa lässt sich eine irreversible Verlagerung des kapitalgesteuerten Wirtschaftens erkennen, die vom klassischen Güterhandel zu den spekulativen Geschäften eines rasenden Finanzkapitalismus geführt hat und damit die kategoriale Wahrnehmung des Gegenstandes Ökonomie und seine Rolle in der Produktion und Stabilisierung sozialer Ungleichheiten grundlegend verändert: Offenbar ist die Grauzone zwischen den materiellen und geistigen Erscheinungen der Ökonomie in einer Spreizung angelangt, wo unklar ist, ob es sich in den zentralen Phänomenen des Gegenstandsbereichs nun um die geistigen Manifestationen der Dingwelt handelt, oder doch eher um ihre ideellen Präfigurationen (Vogl 2021). Kleinste Verlautbarungen können in Sekundenschnelle größte Kursschwankungen erzeugen, ohne dass sich die algorithmischen Mittler, durch die sich solche Schwankungen realisieren, auf der Subjekt-Objekt-Spaltung eindeutig verorten lassen: Sind sie nun die Sprechakte souveräner Subjekte oder eher ein Geflüster, das den technologisch-materiellen Bedingungen auf die Füße folgt? Derartige Fragen sind unentscheidbar geworden und können allenfalls in einer Art paradigmatischer Setzung allenfalls als vorerst gelöst gelten und sind damit stets unsicher.

Auch auf der Seite epistemischer Bemühungen lassen sich ereignisinduzierte Gründe für die zunehmende Beschäftigung mit dem Narrativen anführen, die ebenfalls mit dem Begriff der Postfaktizität zusammenhängen. Die Unfähigkeit der Wirtschaftswissenschaften etwa ein derart schlagendes Ereignis wie die globale Banken- und Finanzkrise abzusehen oder auch nachvollziehbar erklären zu können, nährt die Fundamentalkritik an den Prognosefähigkeiten der statistischen Methode. Offenbar ist sie ziemlich gut in der Berechnung von Standardabweichungen, der Extrapolation des Vergangenen und alle anderen Prozeduren des Weiterdenkens bestehender Routinen und Übereinkünfte, aber eher schlecht im Umgang mit der Kontingenz der Welt. Sollten sich die Randbedingungen des Wirtschaftens nicht konstant halten, werden statistische Verfahren ungeeignet. Dann springt die Logik des Politischen ein und die Wirklichkeit schlägt sich auf die Seite der Wahrnehmungsschemata. Es kommt, wie Vogl schreibt, zur „Konjunktion von Kapital und Ressentiment“ (Vogl 2021). Auf der Grundlage dieser Problematik hat sich neuerdings eine empirische Forschung herausgebildet, die sich verstärkt mit der Diskursivierung des Ungleichheitsphänomens befasst (Bank 2017; Gómez-Jiménez und Toolan 2020; Grisold und Preston 2020; Silke et al. 2019; Smith Ochoa 2020, 2022). Auch wenn der Fokus dabei in der Medienberichterstattung und einer dort konstatierten Diskursmacht von Ökonom:innen und Wirtschaftsexpert:innen (Pühringer 2015) liegt, zeigen diese Studien, dass die Ungleichheitsdebatte nicht anders als jene Kontroversen operiert, die sich als ethische Kontroversen präsentieren und nicht so einfach mit statistischen Werten objektiviert werden können. Der Sonderband widmet sich dieser Perspektive.^3^

## Die politikwissenschaftliche Relevanz von Ungleichheitsnarrativen

Wir gehen von Multidimensionalität aus und begreifen Ungleichheit sowohl als *sozialen Zustand*, der empirisch *festgestellt* oder *erhoben* werden kann, als auch als einen *Erfahrungsgegenstand*, den es zu verstehen gilt. Wir konzentrieren uns zwar schwerpunktmäßig auf die zweite Bestimmung, versammeln in diesem Band aber auch Beiträge, die sich dem ersten Verständnis zuordnen lassen. Die Texte werden auf drei Ebenen behandelt, die uns für eine politikwissenschaftliche Analyse von Ungleichheit und Ungleichheitserzählungen relevant erscheinen.

Die erste Ebene hängt mit der Erwartung zusammen, dass eine Analyse der Erzählungen von sozialer Ungleichheit vor Augen führen könnte, wie das Verhältnis zwischen Wissenschaft, Medien und Politik funktioniert. Hier lassen sich insbesondere die rhetorischen Kapazitäten der Akteure verorten und die Frage, wie sich Narrative als Werkzeuge der politischen Kommunikation spontan entwickeln und im politischen Diskurs zum Einsatz kommen. Einen ersten Aufschlag in diese Richtung bietet *Gert G. Wagner* mit seinem Beitrag zum *Narrativ der unaufhaltsam steigenden Ungleichheit*. Wagner setzt sich am Beispiel der Sozialdemokratie kritisch mit dem Verhältnis von Datenforschung und politischer Programmbildung auseinander und adressiert vor diesem Hintergrund das Problem der Gültigkeit des Ungleichheitsnarrativs. Dabei sieht er besonders die noch neue Datentechnik des *nowcasting* in der Pflicht, um die aus seiner Sicht für die politische Diskursqualität notwendige Validität der Erzählungen erreichen zu können. Forschungsstrategisch ähnlich, aber substantiell anders argumentieren *Irene Becker, Tanja Schmidt* und *Verena Tobsch* im Beitrag *Materielle Teilhabe – Ein mehrdimensionaler Blick auf die „Fakten“ über Verteilung*. Auch sie problematisieren das Problem der Ungleichheitsforschung, die Lebenswirklichkeiten empirisch einwandfrei festzustellen. Allerdings will ihr Beitrag die Korrespondenzaufgabe durch die Berücksichtigung weiterer Datenerhebungsebenen erfüllen, also auf theoretisch-konzeptioneller Ebene lösen, als auf neue Techniken zu verweisen. Ob sich die Erzählungen von Ungleichheit, wie beide annehmen, mit ihren materiellen Bezügen überhaupt korrespondieren lassen, sei hier mal dahingestellt. Beide liefern aber einen guten Einblick in den klassischen Nexus von Wissenschaft und Politik, in der die empirische Forschung die entscheidende Rationalitätsbedingungen politischer Erzählungen darstellt. Aus unserer Sicht könnte eine solche Perspektive künftig stärker von den Möglichkeiten des *Data Storytelling* profitieren.

Eine weitere Ebene hat mit der Vermutung zu tun, dass eine Analyse von Ungleichheitsnarrativen Erkenntnisse darüber verspricht, wie in der Politik mit ausgeprägten normativen und emotionalen Spannungen umgegangen wird, wo doch ihre institutionelle Architektur auf die formale Ausbalancierung politisch organisierter Interessen gerichtet ist. Hier knüpft der Sonderband an die narrative Wende an, die das Erzählen weniger als „Kunstproduktion“, denn als „grundlegendes Verfahren des Menschen, der Welt und dem eigenen Dasein Sinn abzugewinnen“ (Heinen 2007; siehe grundlegender Koschorke 2012). Es geht hier also nicht um den strategischen Einsatz von Narrativen, sondern um die Bildung von Legitimität in einem „Raum der Gründe“, der sich über die Praktik des Erzählens formiert (Gadinger et al. 2014; Yildiz et al. 2018; Smith Ochoa et al. 2021). *Martin Schürz *beginnt diesen Teil mit einem Text *Zum Labyrinth moralischer Gefühle zu Reichtum*. Schürz thematisiert das Verhältnis von Emotion und Narration und argumentiert, dass ein wenig marktvermitteltes Phänomen wie Reichtum eigene Legitimationsformeln hervorbringt, die nicht auf erbrachter Leistung oder Tugendhaftigkeit aufruhen, sondern auf den wohligen Gefühlen, die mit Reichtum allgemein assoziiert werden. Es ist demnach eher eine Art beruhigende Bewunderung oder Sympathie für die Annehmlichkeiten eines glanzvollen Lebens, durch die sich Reichtum im sozialen Raum rechtfertigen kann. Mit dem ästhetischen Aspekt von Gefühlen beschäftigt sich auch *Hans-Ludwig Buchholz* in den *Literarischen Narrativen ökonomischer Ungleichheit* und weist dort auf die Möglichkeiten der Fiktionalität zur Extrapolation fremder Lebens- und Gedankenwelten hin. Am Beispiel einer Interpretation von Hans Falladas *Kleiner Mann – was nun?* geht Buchholz hinter die von Schürz betonte Bewunderung und stößt dort auf den Bereich einer gefühlsmäßig-narrativen Verarbeitung von Ungleichheitserfahrungen, die ohne dem Literarischen der Öffentlichkeit doch in dieser Ausführlichkeit verborgen bliebe. Anschließend geht *Thomas Biebricher* auf die Narrative der Ungleichheit von Neoliberalismus und Neokonservatismus ein und unterzieht sie der Perspektive von politischer Theorie und Philosophie. Ab den späten 1990er-Jahren, so Biebricher, wurden im US-amerikanischen Diskurs neokonservative Narrative dominant, die die Gründe für die sozioökonomischen Ungleichheiten nicht mehr strukturalistisch deuteten, sondern dazu übergegangen sind, ihre Gründe in die von Ungleichheit betroffenen Subjekte zu verlagern. Sozialpolitisch seien sie deshalb allenfalls noch über eine *tough love* zu erreichen und Biebricher fragt, ob sich eine vergleichbare Verschiebung auch für den deutschen Kontext identifizieren lässt.

Die Beiträge des zweiten Teils weisen schon darauf hin; eine Narrativanalyse kann wichtiges über die ästhetischen Praktiken des politischen Rechtfertigens in Erfahrung bringen. Schließlich ist das *Was* des Gesagten auch immer eine Frage seines *Wie*. Der dritte Strang der Beiträge setzt sich deshalb intensiver mit der empirischen Frage nach den Effekten von Ungleichheitsnarrativen auseinander. Der Teil beginnt mit einem Text von *Johanna Kuhlmann* und *Sonja Blum*. Sie greifen darin *Geschichten vom Geben und Nehmen* am Beispiel der politischen Debatten um Kurzarbeit und Elterngeld auf und fragen, wie dort Zielgruppen konstruiert werden und welche Legitimierungsperspektiven damit entstehen. Ähnlich geht auch *Marlon Barbehön* in seinem Beitrag *Die Vermittlung der Ungleichheit* vor. Mehr auf den Aspekt der Symbolizität eingehend greift Barbehön die erzählerischen Transformationsleistungen auf, die aus bloßen Unterschieden soziale Ungleichheiten machen. Am Beispiel von „Mittelschicht“ geht er auf zwei Aspekte des Narrativen ein; auf den der Komplexitätsübermittlung einerseits und auf die Konstruktion eines beweglichen Referenzwertes („Mitte“), an dem sich unterschiedliche Ungleichheitsvorstellungen justieren lassen andererseits. *Matthias Diermeier* und *Judith Niehues* thematisieren die *Ungleichheits-Schlagzeilen auf Bild-Online*. Sie nehmen eine quantitative und qualitative Sentiment Analyse vor, um herauszufinden, ob es sich dabei um ein *Sprachrohr der Werthierarchie* handelt. Auch *Hendrik Theine* und *Andrea Grisold* nehmen Medien zum materialen Gegenstand von Ungleichheit, befassen sich aber aus polit-ökonomischer Perspektive mit den Berichten zur *Vermögens- und Erbschaftsbesteuerung in Deutschland* in sieben Tages- und Wochenzeitungen. Dieser Teil des Bandes schließt mit einem Beitrag von Natalie Rauscher und Maren Schäfer zu* Kommunikationsstrategien und Ungleichheitsnarrative des linken Diskurses im Umfeld der Demokratischen Partei der USA*. Mittels einer korpusgestützen Frame-Analyse zeigen die Autorinnen, dass der *American Dream, *der sich als die Diskursformation einer um die Legitimationskraft der Reichtumsbewunderung kreisenden politischen Kultur rekonstruieren lässt, unter Druck gerät und derweil einer stärkeren Systemkritik ausgesetzt ist.

Der Sonderband soll die sozialwissenschaftliche Auseinandersetzung mit Ungleichheitsnarrativen anregen. Wenngleich mit den drei skizzierten Relevanzebenen doch unterschiedliche, teilweise sich widersprechende Narrativverständnisse verbunden sind und das Spektrum zwischen strategischem Gebrauch und kultureller Praktik doch überaus groß ist, halten wir es hier für sinnvoll, dieses weite Spektrum in der Frage nach der Multidimensionalität der Narrative sozioökonomischer Ungleichheit beizubehalten: Welche Erzählmuster dominieren die Ungleichheitsdebatte in Deutschland und im Ausland? Wie verhalten sich die Akteur:innen der Ungleichheitsdiskussion zur Umstrittenheit der Fakten? Welche alternativen Formen der Gewissheitserzeugung lassen sich ausfindig machen, wie werden sie in den verschiedenen Diskursfeldern der Politik wirksam, von welchen „epistemischen Gemeinschaften“ werden sie getragen und welche politischen Optionen entstehen in diesen Konstellationen? Mit diesem Impuls wird die Ungleichheitsforschung, die sich nach wie vor überwiegend auf die Quantifizierung der Güterverteilung und die Verfeinerung der Erhebungsmethoden beschränkt, mit einer zunehmend an Bedeutung gewinnenden Perspektive konfrontiert, wonach die politische Sprache nicht nur eine instrumentelle Funktion ausübt, sondern mittels Erzählungen auch eine konstitutive Bedeutung für die Konstruktion von politischen Wirklichkeiten besitzt. 

## Footnotes

1 Quantitative Daten nehmen als vermeintliche Ausdrücke von Ist-Zuständen eine zentrale Rolle in der Ungleichheitsdebatte ein. Trotz ihrer methodischen und konzeptionellen Probleme genießen statistische Standardmaße wie der Gini-Koeffizient oder die Armutsquote eine unvergleichbar hohe Wirkmacht im gesamten Diskursgeschehen. In der deutschen Ungleichheitsdiskussion erweisen sich hierbei Institute wie das DIW, das IW Köln, das WSI und das ifo als besonders produktiv in der Schaffung einer empirischen Datengrundlage.

2 In diesem Zusammenhang formuliert Piketty (2020, S. 1273) das Marx’sche historisch materialistische Leitmotiv um: „Die Geschichte aller bisherigen Gesellschaft ist die Geschichte des Kampfs der Ideologien und der Suche nach Gerechtigkeit. Es kommt, anders gesagt, in der Geschichte ganz entscheidend auf Ideen und Ideologien an.“

3 Hintergrund des Sonderbands sind ein interdisziplinärer Workshop „Narrative ökonomischer Ungleichheit“ sowie eine Abendveranstaltung „Reichtum, Armut und Demokratie“ mit Moderatorin Ulrike Winkelmann (taz Chefredaktion), Prof. Dr. Miriam Rehm (Universität Duisburg-Essen), Dr. Gregor Gysi (die LINKE), Dr. Taylan Yildiz (Universität Duisburg-Essen) und Dr. Hans-Peter Klös (IW Köln). Beide Veranstaltungen, die als Abschlussereignis des Hans-Böckler-Stiftung Forschungsprojekts „Politikmanagement im Ungleichheitsdiskurs“ organisiert wurden, fanden am 03.12.2019 an der NRW School of Governance der Universität Duisburg-Essen statt.
